# Harnessing the HDAC–histone deacetylase enzymes, inhibitors and how these can be utilised in tissue engineering

**DOI:** 10.1038/s41368-019-0053-2

**Published:** 2019-06-10

**Authors:** Liam Lawlor, Xuebin B. Yang

**Affiliations:** 1 Department of Oral Biology, University of Leeds, Wellcome Trust Brenner Building, St. James’s University Hospital Leeds, LS9 7TF UK; 2 Doctoral Training Centre in Tissue Engineering and Regenerative Medicine, Institute of Medical and Biological Engineering, School of Mechanical Engineering, University of Leeds, Leeds, UK

**Keywords:** Stem-cell biotechnology, Stem-cell research

## Abstract

There are large knowledge gaps regarding how to control stem cells growth and differentiation. The limitations of currently available technologies, such as growth factors and/or gene therapies has led to the search of alternatives. We explore here how a cell’s epigenome influences determination of cell type, and potential applications in tissue engineering. A prevalent epigenetic modification is the acetylation of DNA core histone proteins. Acetylation levels heavily influence gene transcription. Histone deacetylase (HDAC) enzymes can remove these acetyl groups, leading to the formation of a condensed and more transcriptionally silenced chromatin. Histone deacetylase inhibitors (HDACis) can inhibit these enzymes, resulting in the increased acetylation of histones, thereby affecting gene expression. There is strong evidence to suggest that HDACis can be utilised in stem cell therapies and tissue engineering, potentially providing novel tools to control stem cell fate. This review introduces the structure/function of HDAC enzymes and their links to different tissue types (specifically bone, cardiac, neural tissues), including the history, current status and future perspectives of using HDACis for stem cell research and tissue engineering, with particular attention paid to how different HDAC isoforms may be integral to this field.

## Introduction

There is a largely unmet clinical need for the repair and regeneration of human tissues and organs. Due to the limitations of conventional clinical therapies, tissue engineering, a multidisciplinary area of research, has gained prominence, as it is likely to offer novel solutions to healthcare problems. Some early success has been achieved in transplanting relatively simple tissues or organs such as the trachea^1^ and the bladder.^2^ These early accomplishments demonstrate the massive potential of utilising tissue engineering approaches to restore tissue and organ function, although much further research is needed, especially for more complicated organs and tissues.

Stem cells are an exciting cell source, with the potential to be differentiated into the specific lineages required for the repair and restoration of functional tissues. Increased reporting of the limitations of current methods of using stem cells in tissue engineering has galvanised research to investigate new methods of controlling stem cell fate. However, effectively controlling stem cell proliferation and differentiation, with minimum side effects, is very challenging.

It has become apparent in recent years that ‘epigenetics’ plays a massive role in cell fate. ‘Epigenetics’ refers to the post-genetic modifications made to DNA sequences and related proteins, where chemical functional groups such as methyl or acetyl are transferred onto the DNA or such related protein (e.g., the histone scaffolds, which DNA is wrapped around). Epigenetic modifications make no changes to the actual base genomic code, and epigenetic changes are potentially heritable.^3,4^ Many diseases, perhaps most notably cancers, have epigenetics as a key mediator.^5–7^

More specifically, research has discovered that modifications to the histone, such as those by histone deacetylation enzymes (HDACs), affect chromatin structure, and thus stem cell properties such as potency and differentiation.^3,8,9^ As such, the epigenetic make-up of a cell may be crucial for cell-based therapies and tissue engineering. For example, inhibition of HDACs has been utilised to improve in vitro expansion methods of human hematopoietic stem cells,^10^ to improve bone differentiation of mesenchymal stem cells (MSCs),^11^ to enhance the efficiency of induced pluripotent stem cell (iPSC) generation,^8^ and to increase the efficiency of cellular therapies.^12^

This review will explore background knowledge of epigenetics, the structure, and function of HDACs and their inhibitors, highlight the current uses of and potential of these compounds in cell-based therapies and tissue engineering and suggest where this field may find itself in the future.

## Epigenetics: post-genetic modifications of DNA sequence

DNA, the set of ‘rules’ which control the cell in its most basic form, exists as the famous double helix, is packed into tight but flexible assemblies.^13^ This flexibility allows constant structural alteration so that DNA can be utilised in transcription, replication, and repair.^14^ The 147 base units of DNA wrap around histone proteins, forming nucleosomes. These can be organised further, with the ever-changing histone epigenome affecting how compact or how relaxed these structures are.^3^

Acetylation is one of the most abundant post-translational modifications, and genome-wide analysis of cells has shown that acetylation is as frequent as phosphorylation.^9,15^ The lysine residues on the histone proteins which DNA wraps around are especially important sites of acetylation. The defined combination of these histone modifications has been termed the ‘histone code’.^16^ The histone code can be “read” by proteins, such as bromodomains, but can also be erased and re-written,^17^ predominantly by two classifications of cellular enzymes; histone deacetylases (HDACs) and histone acetyl transferases (HATs). If bromodomains are an example of acetylation ‘readers’, HATs are the ‘writers’, and HDACs are the ‘erasers’.^15^ HATs mediate the transfer of acetyl groups onto the lysine residues (histone acetylation), which results in more open and more transcriptionally active chromatin structures, as the interactions between the nucleosomes are altered, and the histone tails are released from the linker DNA. HDAC enzymes remove those acetyl groups (histone deacetylation), repressing transcription due to the formation of a more condensed and transcriptionally silenced chromatin.^3^ HDAC inhibitors (HDACis) inhibit the action of HDAC enzymes, resulting in increased acetylation levels in the cell. This may affect the chromatin compaction, linked to the potency, or differentiation potential of stem cells.^9^ Despite the misleading name, containing the word ‘histone’, there are a wide range of non-histone targets of these enzyme.^18^

## The structure and activity of HDACs

There are 18 human enzymes known to have deacetylation activity, conventionally numbered 1–18 (e.g., ‘HDAC8’). Based on molecular phylogenetic analysis of primary structures, their location in the cell and their homology to yeast enzymes RPD3 and HDA1, these 18 HDACs are grouped into four classes,^19^ which can be further subdivided into two categories— classical (classes I, II and IV) (see Tables 1 and 2 for a summary) and sirtuins (class III). The activity of the classical HDACs depends on zinc ions (Zn^2+^), whereas the sirtuins utilise nicotinamide adenine dinucleotide (NAD^+^), a phosphate linked dinucleotide coenzyme. As they have entirely different mechanisms of action, sirtuins fall outside the scope of this review; only the classical HDACs are reviewed.^20^

**Table 1:** Summary of Class I zinc-dependent HDAC isoforms

HDAC	Cellular location	Tissue distribution	Known biological functions
HDAC1	Nucleus^193^	Ubiquitous^194^	Cellular proliferation,^195,196^ and cell cycle regulation and haematopoiesis^197,198^ DNA damage response,^199^ cardiac development^115,119^ Oligodendrocyte, glial cell, synapse and neuronal cell development and function,^146,148,154,155,158,200^ regulating Schwann cells^156,201^ Osteogenic and skeletal muscle development,^99,202^ cartilage formation^43^ Modulating T cell response,^203^ epidermal formation,^204^ adipogenesis^205^
HDAC2	Nucleus^193^	Ubiquitous^194^	Cell cycle regulation,^198^ DNA damage response,^199^ cardiac development^115,117^ Oligodendrocyte, glial cell, synapse and neuronal cell development and function,^146,148,154,155,158,160,206^ regulating Schwann cells^151,201^ Skeletal muscle development,^202^ regulating cartilage structure^43^ epidermal formation,^204^ adipogenesis^205^
HDAC3	Nucleus and cytoplasm^193^	Ubiquitous^194^	Cell cycle regulation,^207,208^ Liver function,^38,209^ cardiac function,^120,121^ osteogenic development,^99^ bone mass and bone formation,^67–70,99^ including osteoclast suppression,^82^ chondroprogenitor differentiation to cartilage^68^ Brain function^162^ and proliferation/differentiation of neural cells^145,208^ hematopoietic stem cell growth^10^
HDAC8	Nucleus and cytoplasm^193^	Ubiquitous^194^	Craniofacial, skull and bone formation in embryonic development^210^

**Table 2:** Summary of Class II and IV zinc-dependent HDAC isoforms

Class	HDAC	Cellular location	Tissue distribution	Known biological functions
IIa	HDAC4	Nucleus and cytoplasm^193^	Brain, heart and skeletal muscle,^211,212^ prehypertrophic chondrocytes,^78^ retina,^213^ neurons^214–216^	Myofibroblast development,^40^ chondrocyte hypertrophy and endochondral ossification,^78^ muscular differentiation,^212^ retinal neuronal function,^213^ regulation of neuronal activity, cell death and survival^214,215^
	HDAC5	Nucleus and cytoplasm^193^	Heart, skeletal muscle and brain,^211,217^ neurons^216^	Differentiation of neural stem cells,^145^ and neuronal activity,^216^ myocardial and endothelial functions,^217^ memory function^218^
	HDAC7	Nucleus and cytoplasm^193^	Thymus,^219^ heart, muscle and lung^211^	In embryonic endothelial cells of developing heart, blood vessels, mesenchyme and myocardial layers of heart and in lung tissue,^118^ role in developing thymocytes,^219,220^ osteoclast activity,^82^ inflammatory macrophages^221^
	HDAC9	Nucleus and cytoplasm^193^	Heart, skeletal muscle and brain^211,222^	Redundant role in heart development,^123^ controls genes affected by motor innervation in muscles^167^
IIb	HDAC6	Mainly Cytoplasm^193^	Muscle,^223^ brain,^166^ heart,^224^ liver,^225^ kidneys,^226^ and teste^227^	Neuroprotection and neurodegeneration,^166^ muscular differentiation,^223^ arterial modelling,^224^ tubulin acetylation, bone mass regulation and immune response modulation,^227^ involved in cellular response to stress,^228,229^ and macro-autophagy,^230^ platelet activation^231^
	HDAC10	Nucleus and cytoplasm^193^	Liver, spleen, kidney,^232^ skin^233^	Expressed in the developing brain with neural oligodendrocyte cells,^168^ melanin production in the skin,^233^ promotes autophagy-mediated cell survival in neuroblastoma cells^234^
IV	HDAC11	Nucleus and cytoplasm^193^	Brain,^168^ heart, skeletal muscle, kidney,^235^ T cells^236^	Influences immune activation versus immune tolerance^236^

## HDACis and specificity

HDACis are typically small-molecular compounds which can bind to and block the action of HDAC enzymes. Some have been isolated from natural sources, such as Trichostatin A (TSA), or designed and synthesised in a laboratory, such as MS-275.^21^

**Fig. 1:**
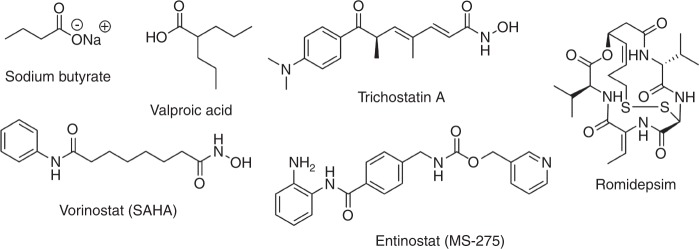
Structures of HDACis commonly found in the literature. Clockwise from top left–Sodium butyrate, Valproic acid, Trichostatin A, Romidepsin, Entinostat (MS-275) and Vorinostat (SAHA)

When HDACis bind to, or block, the active site (e.g., the zinc ion) of HDAC enzymes, they act to block the deacetylation action of the zinc ion. To date, research has largely focused on non-specific HDACi compounds (pan-HDACis); broad spectrum HDACis which target many of the HDAC isoforms.^22^ It is widely accepted that most first generation HDACis, such as Vorinostat (suberanilohydroxamic acid, SAHA) and Romidepsin^23,24^ are relatively isoform unselective.^25^ However, the findings can be conflicting, for example, Bradner and co-workers (2010) found some of these HDACis to be selective for certain HDAC isoforms. Discrepancies can arise due to a lack of knowledge of HDAC structure and problems with screening techniques.^19^ The structure of several commercially available HDACis can be seen in Fig. 1, showing Sodium butyrate, Valproic acid (VPA), Trichostatin A (TSA), Romidepsin, Entinostat (MS-275) and Vorinostat (also known as Zolinza® or suberoylanilide hydroxamic acid, SAHA).

Whilst some pan-HDACis have proven effective drugs,^23,24^ research is beginning to focus on isoform-selective inhibitors. The structural differences between HDAC enzymes mean that HDACis can be designed to be selective for specific enzymes. It has been argued by many that targeting specific HDACs is key to the development of future HDAC therapeutics.^22,25,26^ Table 3 summarises the HDAC class/isoform specificity of some commonly used HDACis.

**Table 3:** Summary of the HDAC class/isoform specificity of commonly used HDACis

HDACi	HDACs targeted	Notes and references
Sodium butyrate	Class I and II	Early pan-HDACi^237^
Valproic acid	Class I and II	Early pan-HDACi^237^
Trichostatin A	HDAC1, HDAC2, HDAC3, HDAC5, HDAC6 and HDAC7 (or “Class I and Class II”)	Conflicting reports: with a low efficacy to HDAC8 in some studies,^238^ or as specific to HDAC1, HDAC3 and HDAC8,^239^ or a high level of targeting many Class I and Class II HDACs^19^
Romidepsin	Class I	FDA anticancer agent used to treat various lymphomas^237^
Entinostat (MS-275)	HDAC1, HDAC2 and HDAC3	Widely reported as an isoform-specific HDACi^237,238^
Vorinostat (SAHA)	HDAC1, HDAC2, HDAC3, HDAC6 and possibly HDAC7	Specificity has conflicting reports–some report as Class I, II and IV, others just HDAC1, HDAC2, HDAC3 and HDAC6.^19^ Multiple reports of just targetting HDAC7 also reported^30^

Some HDACis, (perhaps most extensively Vorinostat), have been explored in clinical trials as cancer treatments. High levels of deacetylation activity have been linked to tumour pathology and utilising HDACis to reduce deacetylation activity which allows cancer suppressing genes to work.^27^ The anti-cancer action of HDACis is also due to many other effects of the compounds on cancer cells, including induction of senescence, activation of apoptosis and mitotic cell death, growth arrest and anti-angiogenesis effects.^28^

Moving on from their applications in oncology, recent publications have shown the huge potential of using HDACis in other fields, such as tissue engineering. The effects of HDACis can be selective for cancerous cells, even at very low doses, and so the concentrations optimised to affect tumour cells typically do not affect normal cells, and their effect on stem/progenitor/normal somatic cells is often unexplored.^24,29,30^

## The potential of using HDACis in tissue engineering

HDACis affect many cellular properties, such as the cell cycle, proliferation rates, gene expression, differentiation potential, accumulation of reactive oxygen species and changes in cell death pathways. This is dependent on the cell type and state, choice of HDACi, and application factors (e.g., exposure time and concentration of inhibitor).^28^

Work has begun attempting to utilise HDACis for the regeneration of different tissues such as bone,^11^ cardiac,^31^ neural/nervous,^32^ adipose,^33^ dental,^34,35^ liver,^36–39^ skin,^40^ pancreas,^41^ muscle^42^ and cartilage.^43^ Also worth noting from a translational tissue engineering perspective are the reports of HDACis demonstrating pain relief,^44–48^ anti-microbial,^49^ immune-modulatory^50–53^ and anti-inflammatory properties.^7,53–55^

Relative to other tissues, a reasonable volume of research has been undertaken into the link between different HDAC enzymes and bone, cardiac and neural/nervous tissues, and further, tissue engineering. Thus, this review article will focus on these three key areas, looking at the history, current status of in vitro and in vivo research, and finally with a nod to future perspectives.

## HDACs and HDACis in bone tissue formation and regeneration

There is still an unmet major clinical need to regenerate bone tissue for fracture repair and the restoration of bone loss due to injury, congenital disorders and degenerative diseases. Researchers currently typically utilise chemicals such as dexamethasone, ascorbic acid and phosphate sources,^56,57^ as well as osteogenic growth factors such as bone morphogenetic proteins to stimulate osteogenic differentiation of stem cells toward bone forming cells.^58^ These osteoblast-like cells can produce collagen matrix and modulate matrix mineralisation and maturation.^59^ However, the current technologies either have limited efficacy, or adverse side effects, resulting in a search for additional or alternative approaches enhancing bone tissue engineering.

### History

Osteoblasts and osteoclasts are the two main cell types responsible for bone formation (osteoblasts), resorption (osteoclasts) and remodelling (both). Research into the effect of HDACis on bone first began in the 1990s, when sodium butyrate (NaB) was found to promote osteoblast bone formation.^60^

Since then, historically, a large number of studies involving HDACis have been to investigate their effect on bone densities, due to initial concerns about the side-effects of HDACis utilised in cancer therapies on bone tissue and density.^61–64^ Some research indicated that SAHA inhibited the growth of bone-related cell populations, such as human bone marrow stem cells, whilst also having the potential to increase osteogenic differentiation in vitro. However, a study found that SAHA did not affect bone mineral density, and could be a promising tool for both oncological and tissue engineering applications.^65^ However, in different animal species, HDACis may affect bone density in different ways.^62^

### HDAC isoforms and their links with bone

It has been suggested that several HDAC isoforms are linked to osteoblast activity, in particular, HDAC3 is thought to be closely linked to bone tissue formation.^66–69^ HDAC3 interacts with Runx2, suppressing osteocalcin production and regulating progenitors to differentiate into osteoblasts.^67,70,71^ During osteoblast differentiation, HDAC3 activity is reduced, resulting in acetylation of histones 3 and 4. This balance of acetylation can be altered in cells by over-expressing or under-expressing Nuclear Factor of Activated T Cells (NFAT) signalling.^72^ Further linking HDAC3 to bone formation, H3, as well as H4, acetylation can promote osteocalcin production, which is essential for bone formation.^73^ In vivo HDAC3 knockout reduced osteoblast levels and increased fatty deposits in bone marrow, seriously compromising skeletal health in mice.^68^ Another study found HDAC3 to be essential for bone maintenance during ageing when knocked down the mouse bone mass was reduced.^69^ Similarly, HDAC1 is downregulated during osteoblast differentiation, making it another potentially interesting bone linked HDAC enzyme to be targeted with specific inhibitors to induce osteoblast differentiation.^74^ Another class 1 HDAC, HDAC8 is known to suppress osteogenic-related genes expression, and inhibition of this with VPA promoted osteogenic expression of rat bone marrow stromal cells.^75^

Jointly HDAC4 and HDAC5 have been found to play a role in osteoblast differentiation.^76,77^ HDAC4 also specifically controls chondrocyte hypertrophy, which is involved in endochondral ossification during bone formation.^78^ Moreover, HDAC6 is linked to the differentiation of MSCs into osteogenic lineages.^79^

Moving on from osteoblasts, both class I and class II HDAC enzymes are required for osteoclast differentiation,^80^ and Romidepsin, which preferentially inhibits HDAC1 and HDAC2, inhibits osteoclastogenesis.^81^ HDAC3 and HDAC7 appear to have opposite effects: suppression of HDAC7 accelerates osteoclast differentiation, while suppression of HDAC3 inhibits osteoclast differentiation.^82^ Similarly, other researchers have suggested that HDAC5 and HDAC6 activity will reduce osteoclast differentiation.^83^

The Wnt signalling pathway is an extensively studied pathway with a key role in the promotion of osteogenic differentiation of MSCs. The Wnt pathway cross-talks with pathways such as the BMP, Notch and Hedgehog pathways, and causes upregulation of osteogenic mediators Runx2, Dl5x and Osterix, as well as suppressing PPARγ. It is likely that increased knowledge of how HDAC enzymes interact with the Wnt pathway will result in improvements in bone tissue engineering protocols.^84^

### Current status of in vitro research for HDACis in bone tissue engineering

Although the specificity of different HDACis is possibly questionable,^19^ it is clear that there are obvious differences in effects between different HDACis on bone-related cells. Reports on the effects of HDACis on bone cells can be conflicting. One study showed that high levels of HDAC acetylation were found to block vitamin D stimulation of osteocalcin production, and thus osteoblast differentiation.^85^ Lee HW and colleagues (2006) showed that NaB and TSA increased osteogenic characteristics in rat cell lines, as well as the upregulation of osteoblast marker genes by NaB.^74^ NaB can also promote osteogenic differentiation of periodontal ligament fibroblasts, and has the added benefit of modulating inflammatory reactions.^55^ Similarly, TSA, MS-275 and VPA have been shown to upregulate pre-osteoblast’s osteogenic gene expression.^86^ However, other reports suggest that TSA may not induce osteoblasts maturation.^60,66^ Another study found that HDAC2-specific MS-275 stimulates bone regeneration both in vitro and in vivo.^87,88^ Thus, the effects of HDACis on cells are still very complex and require further study.

As previously highlighted, several publications demonstrate that HDACis, such as Trichostatin A (TSA) and NaB, inhibit osteoclast differentiation.^60,89,90^ In 2007, T Yi and J Baek reported that TSA could actually cause the death of osteoclasts,^91^ and Kim et al. (2012) suggested that HDAC2-specific inhibitor (MS-275) can reduce bone absorption.^88^ These inhibitory effects on osteoclasts could be utilised to prevent bone loss in inflammatory disease.^92^ Although, contradictorily, it has also been shown that TSA promoted the expression of RANKL, a ligand integral to osteoclast formation, function and survival.^93^ Another HDACi, Largazole was found to increase the in vitro osteogenic potential of C2C12 cells, a murine mesenchymal progenitor cell line, the authors believe this to be due to an increased expression of Runx2 and BMPs.^94^

MSCs can also be derived from dental pulp and have shown great potential for tissue engineering particularly for mineralised tissues such as bone.^56^ VPA has been shown to improve the generation of mineral matrix in dental pulp MSCs, as well as promoting the expression of some osteogenic bone markers such as osteopontin (OPN) and bone sialoprotein (BSP), albeit with a decrease in osteocalcin (OC) expression. It was also demonstrated that shRNA silencing of HDAC2 lead to increased expression of OPN and BSP, but also the downregulation of OC.^95^ TSA has also been shown to promote proliferation and differentiation odontoblasts in DPSCs,^96^ and VPA and TSA have been demonstrated to increase mineralisation of murine dental pulp-derived cell line (MDPC-23) cells.^35^ A recent review of this area has suggested how bone-related markers and HDACs in hPDLs and DPSCs change during odonto/osteogenic differentiation.^97^

It is clear that the choice of HDACi, delivery system and the length of time that cells are exposed to the HDACis may be vital for utilising HDACis in bone tissue engineering.^98^ Several in vitro studies demonstrate the importance of controlling the length of time cells that are exposed to the HDACis, leading to researchers often developing pre-treatment strategies to effect differentiation in in vitro protocols.^99–101^

### Current status of in vivo research for HDACis in bone tissue engineering

In the literature, it is not uncommon for in vivo and in vitro studies to contradict each other. Building on previous work by the group,^101^ one of the key first studies utilising HDACis in bone tissue engineering in 2006 reported that the pan-HDACis TSA and NaB increased osteogenic differentiation of MSCs in vitro and ex vivo, however, these results could not be recreated in in vivo conditions.^11^

In 2010, Hung HM et al. observed an increased osteoblast differentiation when the α-calcium scaffolds with HDACis TSA and NaB were placed in rat bone defect models.^98^ Elsewhere in both an in vitro and in vivo study, HDACis NaB and VPA have been used in combination with reduced oxygen environments and adipose-derived stem cells to achieve the regeneration of bone tissue.^100^ Backing up their in vitro findings, Lee et al. (2011) found that when collagen scaffolds soaked in Largazole solution were implanted in mouse models, there was improved bone formation.^94^

## HDACs and HDACis in heart tissue formation and regeneration

There is a great demand to be able to develop cardiac tissue engineering and regeneration therapies, as heart disease is a major killer worldwide.^102^ It has been suggested that HDAC proteins are key in cardiac tissue development and repair,^103^ as well as the prevention of degenerative cardiac diseases.^104^ For example, HDAC activity has been linked to ischaemic injury, which kills 1 million Americans a year.^105^ HDACis have potential as drugs for treatments of cardiac diseases,^106–110^ as well as in improving cardiac tissue engineering,^31,104,111^ although there is a complicated epigenetic landscape to be understood with each indicated disease associated with the heart.^112^

### History

Cardiac tissue engineering is challenged with selecting a suitable cell type and obtaining and maintaining enough viable cardiomyocytes from stem cell populations. Currently, chemicals such as 5-azacytidine, or other methods such as the coculture of MSCs with mature cardiac muscle cells have been utilised, in an attempt to control stem cell differentiation.^102,113,114^ However, these methods have yielded inconsistent results. Some studies show that there may be a link between HDAC enzymes and cardiac differentiation, therefore a potential to utilise HDACis for cardiac tissue engineering and regeneration.^31,104,111^ Also, such processes are very complicated, and research is far from being able to create functional cardiac tissue.

### HDAC isoforms and their links with cardiac tissue

HDAC1 knockout mice die early in embryogenesis at embryonic day 9.5 due to cardiac defects. HDAC2 deletion leads to survival to the perinatal stage (shortly after birth), but a subsequent death due to a variety of cardiac defects. Cardiac tissue-specific deletion of HDAC1 or HDAC2 alone resulted in no effect on the heart, but deletion of both together resulted in cardiac defects.^115^ Some reports suggested that activity of the class IIa HDACs (HDAC4, 5, 7 and 9) suppress cardiac hypertrophy (heart wall thickening)^116^ whereas class I enzymes (HDAC1 and 2) promote hypotrophy.^117^ Knocking out HDAC7 leads to vascular fatalities in mice.^118^

During coculture with cardiomyocytes, expression of HDAC1 decreases in bone marrow MSCs. When HDAC1 was knocked down in these cells, the expression of cardiomyocyte related genes was then significantly increased, further indicating the key role of HDAC1 in the differentiation down this lineage.^119^ In both mice and humans HDAC3 is upregulated during endothelial differentiation, and HDAC7 was upregulated during smooth muscle differentiation.^103^ HDAC3 is a regulator of cardiac myocyte proliferation during cardiac development in mice^120^ and, also in mice, is essential to the maintenance of cardiac function and cell metabolism.^121^ HDAC4, and HDAC5 in particular are known to be highly expressed in the heart tissue.^122^ Individually knocking out HDAC5 and HDAC9 caused mice to become sensitive to cardiac stress signals, and knocking down both resulted in severe defects, suggesting overlapping functions in cardiac tissue development.^123^

To date, the vast majority of reports studying HDAC isoforms and heart tissue have been carried out in mouse models; however, there are obvious differences between the mouse and the human.^124^ Different methods of assessing the role of HDAC knockdown may also affect results.^125^ Thus, the findings of these studies may not represent the true role of HDACis on human heart disease and development.

### Current status of in vitro research for HDACis in cardiac tissue engineering

TSA is the most studied HDACi in this field; along with SAHA, it has been found to have a strong effect on cardiogenic induction of rat MSCs when compared with the conventional 5-azacytidine protocol.^111,126^ Interestingly, pre-treatment of rat MSCs with 5-azacytidine enhances the effect of TSA on MSC carcinogenesis, and improvements were also demonstrated when adding TSA treatment to the coculture of MSCs and neonatal cardiomyocytes protocol.^111^ These researchers suggest that the combination of HDACis with existing methods could be the future of this research.

TSA has been utilised to stimulate the differentiation of human adipose-derived stem cells into cardiomyocytes.^127^ TSA has also been shown to induce other cardiac cell type differentiation (e.g., endothelial and smooth muscle cells) from cardiac side population stem cells.^128^ In 2010, Kaichi et al. reported that TSA could reduce inconsistencies and greatly improve the quality of mouse cardiomyocytes differentiated from mouse iPSC lines.^129^ Perhaps even more interestingly, a short term treatment of human or mouse embryonic stem cells (ESCs) with TSA can dramatically promote the differentiation of these ESCs into cardiomyocytes, and further increase the cardiac function of the resulting cardiomyocytes.^130–132^ Furthermore, the use of TSA with mouse embryonic carcinoma stem cells promoted cardiac development; however, the upregulation of HDAC4 specifically decreased cardiomyogenesis.^133^

Aside from TSA, Chow and co-workers (2013) used VPA to promote the maturation of human ESC-derived ventricular cardiomyocytes. Interestingly, they also suggest that this may be context dependent and that the developmental stage of the cells being treated could influence the outcome.^124^

### Current status of in vivo research for HDACis in cardiac tissue engineering

There has been limited in vivo validation of the potential of HDACis in cardiac repair. Lee et al. (2007) proved that VPA and tributyrin had a positive effect on the remodelling of damage rat ventricles.^12^ Further work has shown TSA can improve ventricular function recovery in a defect model, which was found to be c-kit signalling dependent.^134^

## HDACs and HDACis in the brain, and nervous tissue formation and regeneration

The typical mammalian nervous system of the body is made up of several distinctly different tissue types: the brain and the spinal cord make up the central nervous system (CNS), separate from the peripheral nervous system (PNS), which exists to relay information to and from the CNS.^135^ Researchers are struggling to develop effective strategies to heal injuries, or generate tissue from both the CNS and the PNS. Investigations have shed some light onto key HDAC enzymes in these tissues and cell populations, often drawing on the fact HDACis (particularly VPA) have historically actually been used for the treatment of various neurological diseases.^136,137^

### History

HDACis have been used in the treatment of neurodegenerative diseases since the 1970s, primarily used to prevent neuron damage.^138^ Although VPA earned FDA approval in 1987 for its use as an anticonvulsant and mood stabiliser,^21^ it was not until 2001 that histone deacetylation inhibition was identified as the mode of action of VPA, resulting in its reclassification as a HDACi.^139,140^

Tissue engineering of these very complicated tissues is a relatively undeveloped area (especially when compared to the previously discussed bone and heart tissues), and differentiation protocols are still being refined and developed. For nervous tissue engineering, current research mainly focuses on the use of nerve guide conduits in combination with different growth factors.^141^ Hu et al. (2012) reported that there might be potential for epigenetic control to be exploited in nervous tissue engineering.^142^ However, the CNS has a much lower regenerative capacity than the PNS, and current therapies focus mainly on preventing neurodegeneration, rather than actual full regeneration. Regeneration with stem cells and biodegradable and bio-absorbable materials such as polyethene, collagen, gelatin, and chitosan have been explored in the brain and spinal cord, but there is a vast volume of work to be done.^143^

### HDAC isoforms and their links with neural and nervous tissue

HDAC enzymes play a key role in neural cell formation in mouse ESCs.^144^ Some HDACs (e.g., HDAC1, HDAC3, HDAC5 and HDAC7) are highly expressed in neural stem cells but are downregulated as neuronal cells differentiate, and therefore are thought to play a role in neuronal differentiation.^145,146^

More specifically, HDAC1 induces the differentiation of retinal progenitor cells and motor neurons in zebrafish development.^147^ HDAC1 and HDAC2 are linked to neural stem and progenitor cells and synaptic formation, but only HDAC1 is expressed in fully differentiated glia.^146,148^ However, HDAC2 is upregulated in neuronal differentiation, while HDAC1, HDAC3, HDAC5 and HDAC7 are downregulated.^149–153^ In mice, HDAC1 and HDAC2 activity are required for oligodendrocyte differentiation, as well as Schwann cell development.^154,155^ They are also regulators of myelination in the peripheral nervous system.^156^ However, other researchers report that, in general, HDAC activity inhibits the differentiation of oligodendrocytes to neurons.^157^ HDAC2 activity may be related to the differentiation of neural progenitors,^151,153^ and can inhibit astrocyte differentiation.^154^ Knockout of both HDAC1 and HDAC2 in mice neuronal precursor cells leads to cell abnormalities, disorganisation and postnatal mouse fatality.^158^ Individually knocking down HDAC1 or HDAC2 also affects neuronal development of mice.^159,160^

HDAC3 is key to neuronal survival, control of apoptosis, and is linked to neural disease,^161^ and memory function.^162^ HDAC3, together with HDAC1 and HDAC2, has been linked to cortical cell differentiation.^154^ HDAC8 is not found to be expressed in normal cells from the central or peripheral nervous system, but is linked to related cancers, notably childhood neuroblastoma, so could be a promising target in this field.^26,163^

Caution should be exercised when exploring HDACis in this field, as it has been demonstrated that targeting class I HDACs with inhibitors in human oligodendrocyte progenitor cells leads to a reduction in progenitor recruitment via proliferation arrest, and inhibition of differentiation into oligodendrocytes. Consequently, Conway and co-workers emphasis that it is also important to realise that there are subtle differences between species in regulation of HDAC activity.^157^

Of the class II HDACs, HDAC4 is key, to neuronal activity, and highly expressed in the brain, in neurodegenerative disorders, and HDAC4 activity is thought to provide the neurons protection from cell death by preventing abortive cell cycle progression.^164^ HDAC5, HDAC6 and HDAC7 are all linked to neural diseases, such as Huntingdon’s disease, protein aggregated diseases and to neural mitochondrial-related diseases respectively.^163^ HDAC5 mediated pathways are linked to early-stage neural cell fate.^165^ HDAC6 has a complicated role in these systems with both a neuroprotective and neurodegenerative role.^166^ HDAC9 is highly expressed in the brain and is proposed to be important in the formation of nerve synapses.^167^

Interestingly, the only class IV HDAC, HDAC11, is barely expressed in precursors to neural cells, but largely features in mature neurons, and therefore is thought to be key to postnatal development of neurons.^168^

### Current status of in vitro research for HDACis in neural and nervous tissue engineering

Neural stem cells were first isolated and reported in the early 90s,^169,170^ and subsequently several related other distinct cell populations have since been discovered.^171,172^ Due to the importance of different HDAC isoforms in neuronal and nervous tissue development, the use of specific HDACis could be an especially promising approach for improving the current approaches in tissue engineering. However, so far, only limited studies into the effects of HDACis on relevant neuronal cells have been undertaken.

As Valproic acid (VPA) obtained FDA approval for treating neurological diseases in the 1980s, there has been a relatively large volume of work on the effects of VPA on cells of these systems.^173^ VPA can induce neuronal differentiation of adult rat hippocampal neural progenitor cells whilst inhibiting glial cell differentiation; favouring neuronal differentiation.^32^ VPA can alter cell function and microglial phenotype of fully developed human glial cells, although it does not result in apoptosis as shown in rodent cells.^174^ In a different study, VPA was shown to enhance rat cerebral cortex neural progenitor cell neural differentiation both in vitro and in vivo.^175^ Moreover, VPA has been shown to control oligodendrocyte progenitor differentiation.^150^ VPA induces proneural factors in rat cells, specifically it has been shown that early stage progenitor cells are more sensitive to the effects of HDAC inhibition, which is thought to be linked to H4 acetylation.^176^ Juliandi and co-workers (2012) reported that VPA treatment enhanced the differentiation of mouse-derived ESCs into neuronal lineage layers.^177^ However, VPA has also been demonstrated to inhibit proliferation, cell “stemness” and the ability to form neurospheres of mice adult subventricular zone cells, a separate neural cell population.^178^ Finally, it has been demonstrated that VPA increases axonal growth of neurons in vitro.^179^

Similar to VPA, TSA has also been utilised to induce mouse neural cells (in minimal supplement media) to express some of the properties of fully functioning neurons.^180^ In a separate study, Siebzehnrubl et al. (2007) found that the HDACis MS-275, M344 and SAHA induced the neuronal differentiation of mouse forebrain precursor cells.^181^ Also, HDACi treatment has been combined with extrinsic transcription factors to promote mouse neural cells to differentiate into dopaminergic neurons.^182^ When treated with HDACis, mouse cells showed increased plasticity, reversed lineage commitment and upregulation of 13 genes associated with neural stem cell states, e.g., Sox2.^183^ Interestingly, HDAC1 and HDAC2 inhibition can be detrimental to oligodendrocyte differentiation.^157,183^ However, in comparison, HES5 (a notch regulator) was not upregulated in human cells. Thus, once again in this field, it is important to note that not all animal cell models may be directly translatable into human models.^157^

### Current status of in vivo research for HDACis in nervous tissue engineering

Unsurprisingly, given the limited number of in vitro studies, there is only a handful of in vivo studies specifically related to tissue engineering and/or regeneration. An in vivo model of the aforementioned in vitro study demonstrated that VPA regulates differentiation and proliferation of rat cerebral cortex neural progenitor cells in the cerebral cortex of developing rat embryos.^175^ VPA has also been proven to reduce cell apoptosis and increase locomotion recovery in a rat spinal cord injury model.^184^ VPA has also been shown to increase the differentiation of NSCs into neurons, not glial cells, along with improved limb function, in mouse spinal cord injury models.^185^

## A brief discussion of the role of HDACs and HDACis in skeletal muscle

Skeletal muscle tissue formation is also regulated by signalling pathways such as Wnt, BMP and the Class II HDACs (HDAC4, HDAC5, HDAC7 and HDAC9) are highly expressed in skeletal muscle. HDACs play a key role in the epigenetic regulation of embryonic myogenesis and adult muscle regeneration.^186^ For example Class II HDACs are known to directly bind myocyte enhancer factor-2 (MEF2) and consequently repress expression of MEF2-dependent genes.^187,188^ HDAC enzymes and their signalling pathways play a key role in regulating myocyte differentiation and remodelling of muscle tissue.^187,189^ HDAC4, in particular, is necessary for maintaining skeletal muscle homoeostasis and function. And it has been identified as a potential target for treatment of skeletal muscle disorders such as cancer cachexia, amyotrophic lateral sclerosis (ALS) or muscular dystrophy.^190^

## Summary

In these three key tissue areas, it has been demonstrated HDACis could be very useful tools to improve tissue engineering strategies. Since bone tissue engineering has found early success with studies combining MSCs with HDACis,^11,100,101^ there is potential to translate this research into clinical settings. Similarly, for cardiac tissue engineering, the many studies involving TSA is a strong beginning for this field,^108^ and when isoform-specific strategies begin, HDAC4 in particular has proven a potentially interesting target for cardiac tissue engineering.^122^ Regarding neural tissue engineering, most of the studies have explored the use of VPA, which is considered a pan-HDACi. Therefore, it is believed that selective isoform targeting may lead to improvements in this field.^163,191^

Presently, researchers aim to combine suitable cells (e.g., MSCs) with scaffolds, chemical cues (e.g., growth factors) and environmental stimuli (e.g., mechanical stimulation) to general functional tissue, such as new nerves, bone and other muscular-skeletal tissues in dentistry and/or orthopaedics. This can be carried out in vitro before being implanted/transplanted in vivo, where the naturally occurring cells can populate the scaffold and chemical cues aid the growth of the targeted tissues. Therefore, HDACis could be utilised as the chemical cues to improve the efficacy of current tissue engineering techniques.

## Future perspectives

There has been some early success stories utilising HDACis in tissue engineering, but looking to the future, the rise of isoform-specific HDACis may lead this research to new heights. There is still plenty to be said for the classical HDACis; however, the connection between tissues and specific HDAC isoforms should lead to more examples of targeted tissue engineering scenarios.

The current limitations of the technologies must be considered:

- a lack of understanding of the pathways HDAC enzymes themselves are involved in, and the effects of these enzymes on cellular properties and behaviour;
- a fundamental lack of knowledge of the function of individual HDAC enzymes, in different cell/tissue types;
- the mechanism of action of many HDACis, i.e., how pan-HDACi’s function, what HDAC isoforms each actually inhibits, and therefore their true mechanisms of action;
- full optimisation of the desired concentration and treatment period of each HDACi on individual cells, to give the best outcome; and
- the need for extensive in vivo testing in clinically relevant animal models, with particular attention to the side effects, before the translation of any HDACis for clinical therapies in this area.

## Conclusion

In conclusion, the field of HDACis in tissue engineering is still in its infancy compared with cancer research where at least 4 HDACis have achieved clinical approval, with many others in late-stage clinical trials.^192^ As demonstrated herein, easily synthesised small-molecular compounds in low concentrations could massively improve the efficacy of stem cell therapies and tissue engineering, potentially without the risks associated with genome altering therapies. As discussed at length in this review, there is a potential paradigm shift in this field with the rise of isoform-specific HDACis. As more regulatory barriers are cut, due to increased clinical approval of HDACis, the potential to translate these compounds in other filed such as in tissue engineering has been increased.
